# Unilateral and Bilateral Cortical Resection: Effects on Spike-Wave Discharges in a Genetic Absence Epilepsy Model

**DOI:** 10.1371/journal.pone.0133594

**Published:** 2015-08-11

**Authors:** Francesca Scicchitano, Clementina M. van Rijn, Gilles van Luijtelaar

**Affiliations:** 1 Department of Biological Psychology, Donders Centre for Cognition, Donders Institution of Brain, Cognition and Behavior, Radboud University, Nijmegen, The Netherlands; 2 Department of Health Science, School of Medicine and Surgery, University “Magna Graecia” of Catanzaro, Viale Europa—Germaneto, 88100, Catanzaro, Italy; University Paris 6, FRANCE

## Abstract

**Research Question:**

Recent discoveries have challenged the traditional view that the thalamus is the primary source driving spike-and-wave discharges (SWDs). At odds, SWDs in genetic absence models have a cortical focal origin in the deep layers of the perioral region of the somatosensory cortex. The present study examines the effect of unilateral and bilateral surgical resection of the assumed focal cortical region on the occurrence of SWDs in anesthetized WAG/Rij rats, a well described and validated genetic absence model.

**Methods:**

Male WAG/Rij rats were used: 9 in the resected and 6 in the control group. EEG recordings were made before and after craniectomy, after unilateral and after bilateral removal of the focal region.

**Results:**

SWDs decreased after unilateral cortical resection, while SWDs were no longer noticed after bilateral resection. This was also the case when the resected areas were restricted to layers I-IV with layers V and VI intact.

**Conclusions:**

These results suggest that SWDs are completely abolished after bilateral removal of the focal region, most likely by interference with an intracortical columnar circuit. The evidence suggests that absence epilepsy is a network type of epilepsy since interference with only the local cortical network abolishes all seizures.

## Introduction

The neurological syndrome epilepsy is characterized by the presence of recurrent spontaneous seizures although they are manifested in different ways. Absence seizures are commonly, but not exclusively, seen in children between 4 and 12 years old [1]; they are classified as generalized and predominantly nonmotor with impaired responsiveness [2]. The electroencephalographic (EEG) examination records bilateral, synchronous, and symmetrical spike-wave discharges (SWDs) with a frequency of 3–4 Hz on a normal background activity, first described by Gibbs et al. [3]. The search for mechanisms of generation, maintenance and abortion of SWDs typical of absence seizures has been carried out for more than half a century [4], and is still emerging. It is not possible as yet to have a clear picture about all processes and mechanisms involved, also considering that most concepts and theories are gained from different animal models and/or in vitro studies, and cannot be easily verified in humans. In general, the hypothesis that SWDs are generated within the cortico-thalamo-cortical network is widely accepted [5,6,7,8,9,10].

A relatively new theory for the initiation and generalization of absence seizures has been achieved in the genetic absence models with a detailed analyses of perictal local field potentials of a cortical grid on the somatosensory cortex and thalamic depth recordings with a nonlinear association analyses in the WAG/Rij rat and through intracellular recordings combined with local field potentials in different layers of the somatosensory cortex in GAERS [7,11,12]. While the former authors identified a cortical initiation zone in the peri-oral region of the somatosensory cortex, the latter ones showed that cells located in deep layers (layer VI) of the somatosensory cortex show a massive increase in firing already before SWDs onset. This introduced a location refinement of the cortical focus to the subgranular layers.

Also results of recent studies in WAG/Rij rats are in line with the “cortical focus” theory establishing that the deep somatosensory cortex of absence epileptic rat is more excitable than the motor cortex and that this difference was not present in control rats [13]. Next, there is more seizure related pre-SWD activity in the focal cortical region than in the thalamus [14]. Other evidence (pharmacologic and neurochemical) for a cortical hyperexcitable region and network analyses in children with absence seizures has been reviewed recently [8,10,15,16]. The presumed focal origin of the “generalized” SWDs has already led to the exploration of a new experimental therapy such as bilateral local transcranial electrical stimulation of the focal regions [15].

A second issue, relevant from a clinical perspective, is that focal epilepsies can be treated with surgical resection. Surgical resection itself has been demonstrated to be sure and effective for the treatment of patients resistant to pharmacotherapy where there is inadequate seizure control [17]. Surgical treatment has never been considered in patients with refractory types of absence epilepsy and a reference protocol does not exist. However, if cortical focal sites of origin can be identified unambiguously, and its locations allow resection, then the possibility can be considered. Here, surgical resection of the epileptogenic zones on seizure occurrence is evaluated in WAG/Rij rats. By this, a property of focal epilepsies is investigated: if absence epilepsy is considered to be a focal type of epilepsy, then it is hypothesized that unilateral and bilateral surgical resection of the assumed focal region should decrease and abolish SWDs. The study may therefore also contribute to the discussion whether absence epilepsy should be considered as a focal, a generalized type of a network type of epilepsy [10, 11, 12, 15, 18].

## Materials and Methods

### Animals

15 male WAG/Rij rats, age 12 months, body weight 320–370 g, were used as experimental subjects. They were born and raised at the Department of Biological Psychology, Donders Centre for Cognition, Radboud University Nijmegen, The Netherlands. Prior to surgery the rats were housed in pairs (High Makrolon cages with Enviro Dri bedding material and cage enrichment) with free access to food and water and were kept under environmentally controlled conditions (ambient temperature = 22°C, humidity = 40%) in a room with reversed light—dark cycle (light on from 8:00 p.m. to 8:00 a.m.). The experiment, performed in accordance with Institutional and ARRIVE guidelines, was approved by the Ethical Committee on Animal Experimentation of Radboud University Nijmegen (RU-DEC). All efforts were done to keep the discomfort of the animals as minimal as possible; therefore it was decided to do the experiment in fully anesthetized animals.

### Drugs

The experiments were carried out in anesthetized WAG/Rij rats: a combination of the mu opioid receptor agonist Buprenorphine (Vetergesic Multidosis, Ecuphar, The Netherland, solution 0.3 mg/ml) diluted (1 to 5) in saline (0.9% NaCl) was given subcutaneous (s.c.) at a concentration of 0.05 mg/ml in a volume of 1 ml/kg and haloperidol (Haldol, Janssen-Cilag BV Tilburg, Netherlands), at a concentration of 5 mg/ml also in a volume of 1 ml/kg was injected intraperitoneally (i.p.). A combination of a mu opioid receptor agonist and a D2 receptor antagonist was shown to have SWD enhancing effects closely mimicking the physiological SWDs regarding amplitude of the spikes, the intraspike frequency, and the morphology of the spike and waves spontaneous in free moving WAG/Rij and GAERS [19, 20]. Moreover, the same drugs do not elicit SWDs in non-epileptic rats.

#### Surgery and EEG recordings

The experiment was done while the rats were in the stereotactic apparatus. EEG recordings were made with the aid of two tripolar stainless steel electrode sets (Plastic One, Roanoke, VI, USA: MS 333/2). The electrode sets were kept in place by a custom made electrode holder; two out of four active electrodes were placed on the frontal region, coordinates with the skull surface flat and from bregma zero—zero, (AP + 4.2 mm, LF ± 3 mm) and two in the parietal region (AP -6.5 mm, LF ± 4 mm). Ground and reference electrodes were implanted symmetrically over both sides of the cerebellum. These and all following stereotactic coordinates were relative to bregma and according to the atlas of Paxinos and Watson [21]. Two differential EEG recordings were made, one from the right and one from the left hemispheres. EEG signals were allowed to pass between 1 and 100 Hz, digitalized at 200 samples s^-1^, and stored for off-line analysis using Windaq system (DATAQ Instruments, Akron, OH, USA). After the EEG electrodes have been epidurally placed (surgery lasted about 30 minutes), the EEG of the 2 groups was recorded for one hour as base-line (precraniectomy) control. The wash-out period of isoflurane was about 30 minutes, therefore, only the data of the last half an hour were compared between the various phases of the experiment.

### Experimental protocol

The rats were divided in two groups: 9 experimental and 6 control rats. Both groups received the general anesthetic isoflurane (Pharmachemie BV, Haarlem, the Netherlands) in combination with a mixture of the analgesic buprenorphine and the antipsychotic haloperidol during the implantation of the electrode sets, during the removal of the cranium above the somatosensory cortex (experimental and control groups), and during resection of the somatosensory cortex. During the EEG recording isoflurane administration was stopped and rats were anesthetized only via the neurolept-analgesic. The first injection with the analgesic buprenorphine occurred 30 min before the start of the surgery.

The skull was removed (craniectomy) in both the rats of the experimental and control group, cortical resection only in the rats of the experimental group.

#### Craniectomy

The dorsal part of the skull was exposed. A piezosurgery device (Mectron, Carasco, Italy, rounded/straight tip, mode power high; pump 2; frequency 25 kHz) was used to remove part of the cranium on the right and left side. The piezosurgery tip was constantly irrigated with ACSF. Special care was taken in order to avoid perforation of the dura or to cause any mechanical injury to brain tissue.

**Fig 1:**
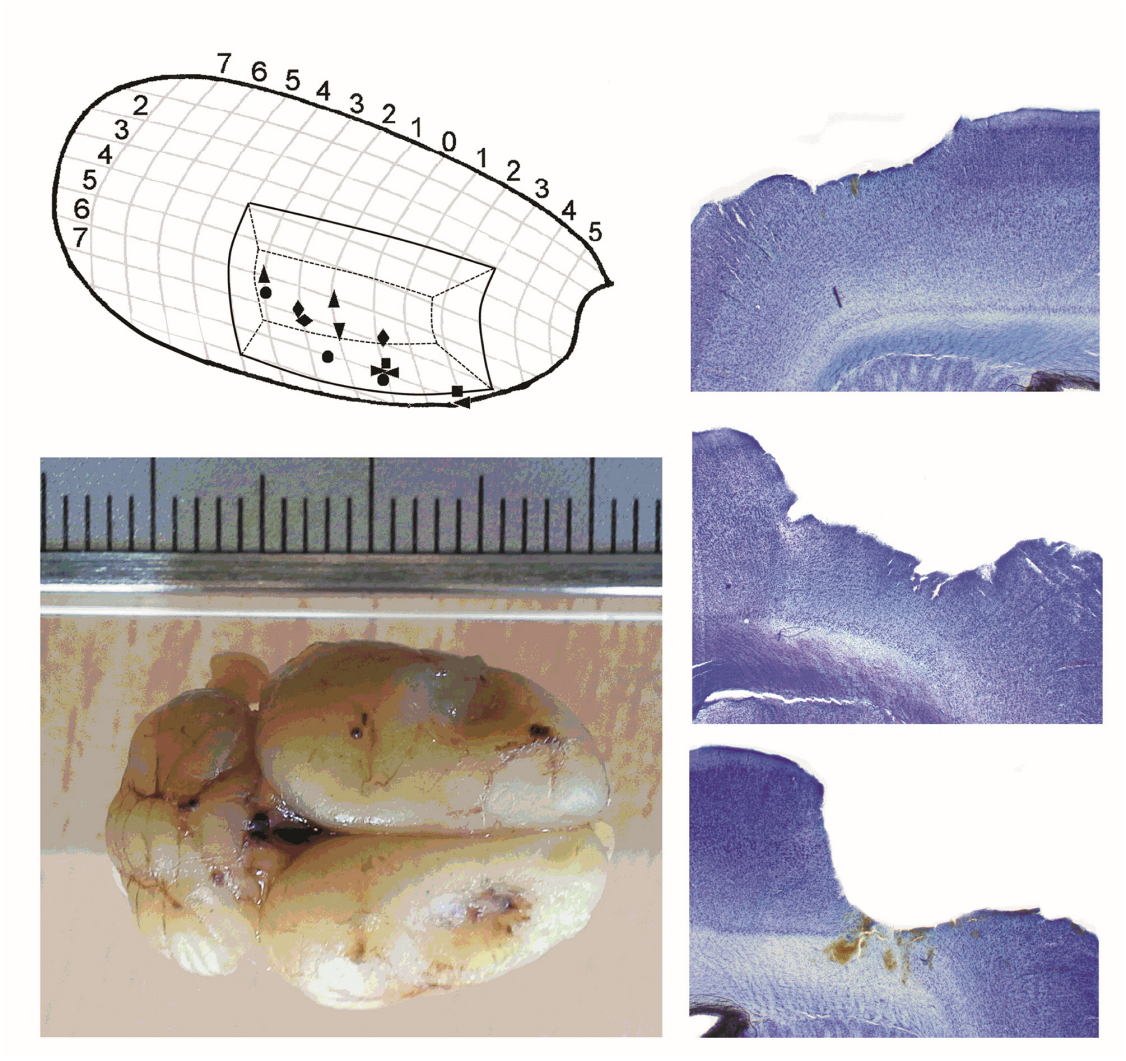
Cortical lesions. Top, left: Representation of the cortical resected regions in the right hemisphere, against the location of established focal zones of 8 WAG/Rij rats (after Meeren et al., 2002) and Bottom-left an example of a WAG/Rij brain with a global impression of the bilateral resected areas supposedly containing the bilateral focal regions. Right side: Example of different cortical lesions. Top: left hemisphere lesion restricted to layer I, II/III. Middle: right hemisphere lesion of layer I, II/III and some damage in layers IV/V. Bottom: right hemisphere lesion layers I-VI.

The coordinates used for craniectomy are indicated in Fig 1; they enclose the cortical region with the assumed focal area in the somatosensory cortex, as was earlier established in this model [11]. The coordinates of the resected cranium were (with bregma at 0,0) from AP 3.0, L ± 2.4 till 5.4 mm to AP -4, L ± 3.2 mm till 6.2 mm. First the right cranium was removed, in the experimental group this was followed by removal of a trench of the ipsilateral somatosensory cortex. This was followed by craniectomy of the left hemisphere and a similar trench of the left hemisphere.

#### Cortical resection

Fig 1 shows the resected part of the somatosensory cortex superimposed on the graph of Meeren et al [11] in which the foci were found as was determined in 8 individual rats in a mapping study with a cortical grid. The aim was to resect grey matter until the corpus callosum was visible. The removed area is the region in which local injections of phenytoin and ethosuximide reduce SWDs [22,23,24], with an increased expression of a subtype of Na^+^ channels [25], a reduction of HCN channels [26] and a high cortical excitability established *in vivo* in free moving WAG/Rij rats [13].

**Fig 2:**
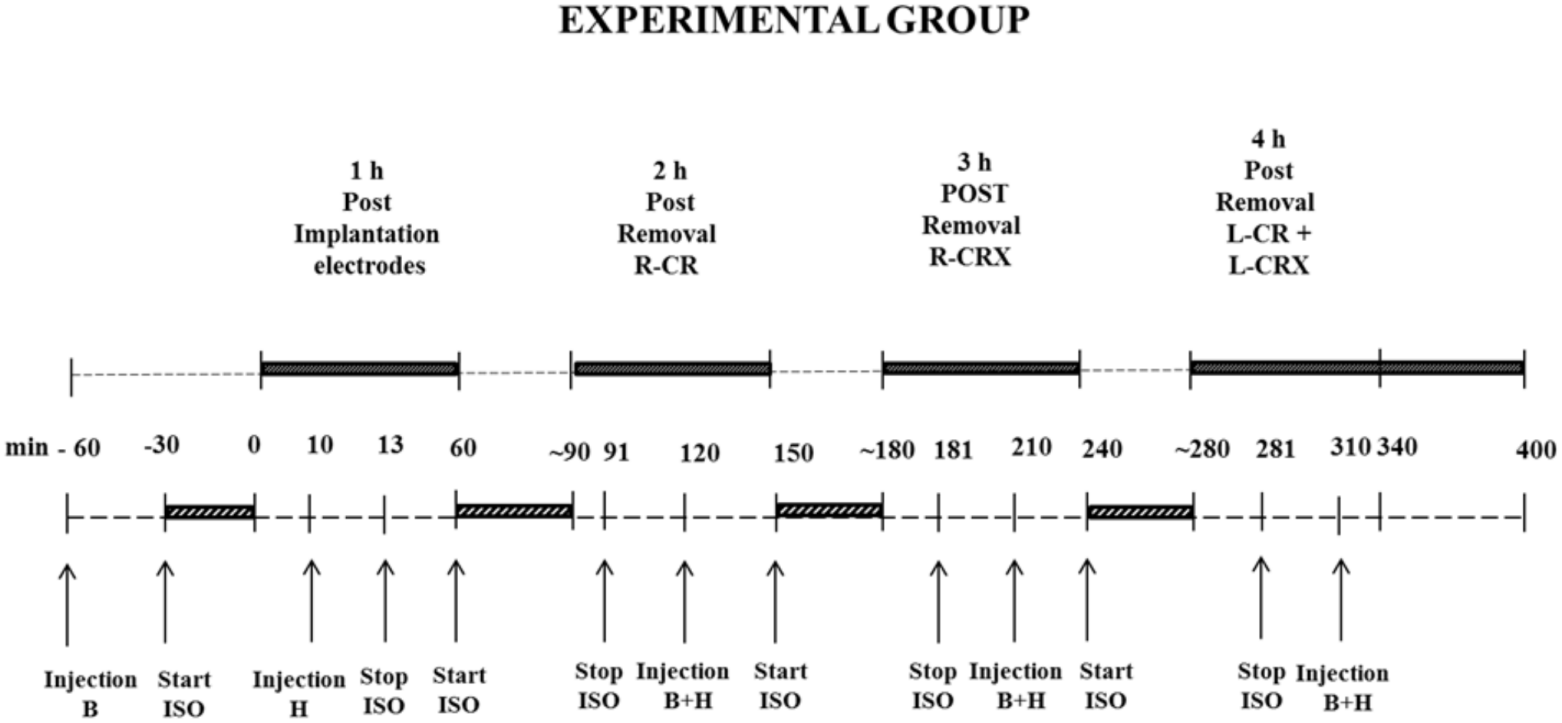
EEG recordings of experimental group. Black rectangle = EEG recording; Grey diagonal striped rectangle = time surgery; B = Buprenorphine; H = Haloperidol; ISO = Isoflurane; min = minutes; L-CR = removal of left cranium; R-CR = removal of right cranium; L-CRX = removal of left cortex; R-CRX = removal of right cortex.

**Fig 3:**
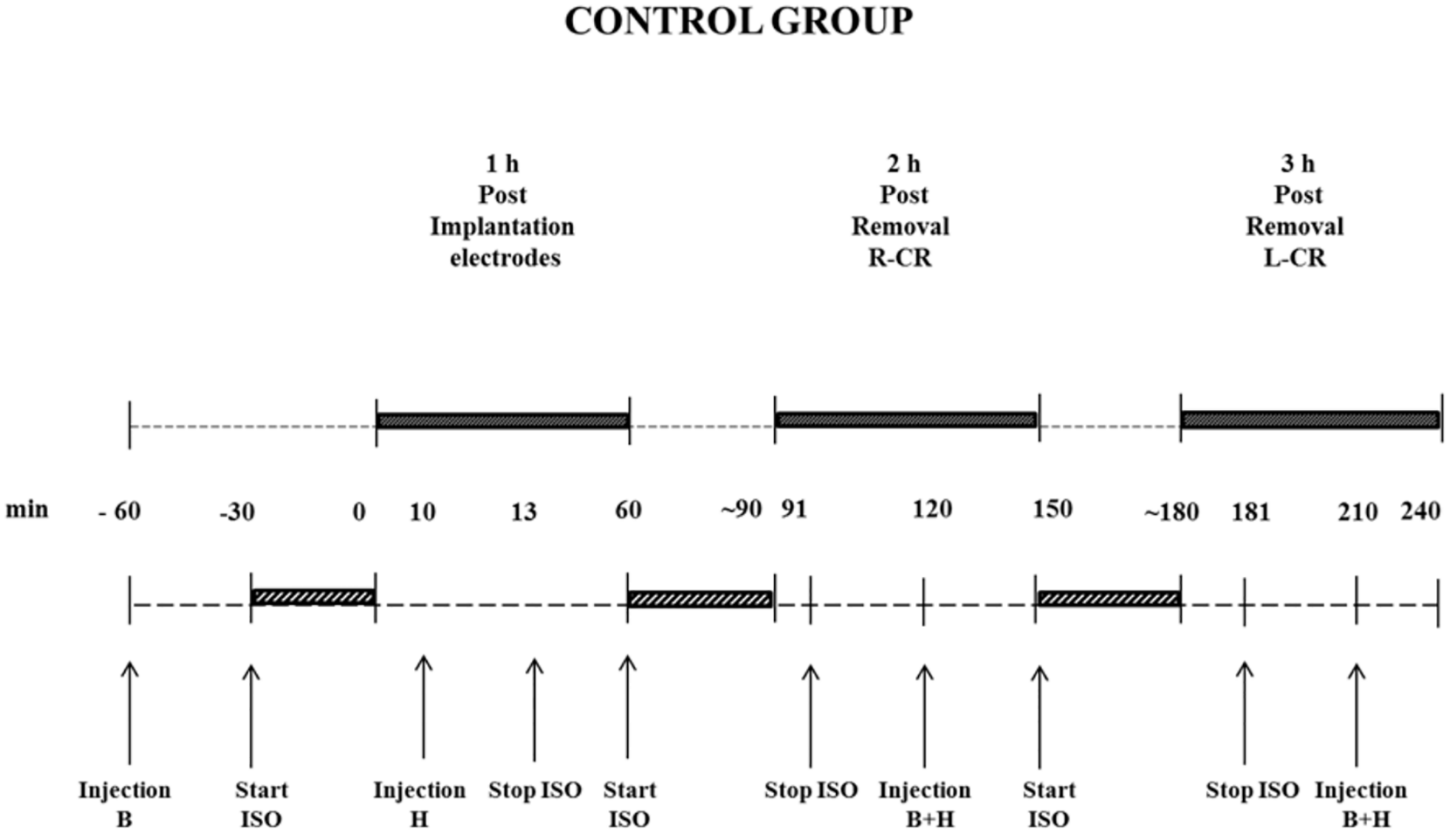
EEG recording of control group. Black rectangle = EEG recording; Grey diagonal striped rectangle = time surgery; B = Buprenorphine; H = Haloperidol; ISO = Isoflurane; min = minutes; L-CR = removal of left cranium; R-CR = removal of right cranium.

The injection and recording times of both the experimental and control groups are presented in Figs 2 and 3. Subsequent injections of haloperidol (half-life 2 hours [27]) and buprenorphine (half-life 2–4 hours [28]) were given in order to maintain an appropriate depth of anaesthesia, which was regularly monitored via toe pinch reflexes. After the end of the day, the rats were still anesthetized, they were given an overdose of ketamine (100mg/ml)/xylazine (20 mg/ml), systemically administered, without any sign of adverse reaction. Next the brains were quickly removed.

The resection of the somatosensory cortex was performed under a binocular stereo microscope (Euromex, Arnhem, Holland); the dura was incised by a sharp steel blade (length = 3 mm) to expose the cortical zone. A flat steel blade with a cutting surface 2 mm length × 1.5 mm width was used to make cortical incisions, next cortical tissue within the target area was removed by a steel curved blade, while contemporarily blooding was stanched with cotton. The depth of the incisions was determined by the coordinates above described. Considering the poor discriminability between grey matter of the cortex and white matter of the corpus callosum, the size of the lesioned area including its depth was subsequently verified by histological verifications. Body temperature was monitored and kept at 37°C via a heating pad, other vital parameters such as respiration were monitored continuously.

#### EEG recordings and analyses

Rats of the experimental group were recorded for 5 hours (Fig 2). In the 10 min of the first hour of recording, rats were still given isoflurane anaesthesia, they had received buprenorphine 30 min before starting the surgery, after successful implantation of the EEG electrodes haloperidol was administered (i.p.), and isoflurane was stopped 3 minutes after haloperidol injection. Isoflurane induced an EEG with burst suppression without any SWDs, the combination of the neurolept analgesic anaesthesia mixture allowed the occurrence of SWD after isoflurane was washed-out. The combination of high dose of haloperidol (5 mg/kg) with buprenorphine increases immobile behaviour but it also increases the analgesic effects of buprenorphine [29]. The second hour was recorded following right side craniectomy at the right side under influence of the mixture while isoflurane anaesthesia was stopped 1 min after the beginning of recording. The third hour of EEG recording followed the resection of somatosensory cortex right side under influence of the mixture while isoflurane anaesthesia was stopped 1 min after the beginning of recording. The fourth hour of EEG recording was after left sided craniectomy and left-side cortical resection and under influence of the mixture. The fifth hour was an extra hour, to make sure that SWDs did not return, again no isoflurane was administered and rats were still anesthetized by the mixture. The behaviour of the rats was constantly monitored by a biotechnician (SMH).

Rats of the control group (Fig 3) were recorded under identical anaesthetic conditions; in the 10 min of the first hour of recording the rats were still given isoflurane, they had received buprenorphine 30 min before starting the implantation of the EEG recording electrodes, after successful implantation of the EEG electrodes haloperidol was administered, and isoflurane was stopped 3 minutes after haloperidol injection, similar as rats from the experimental group. The second and third hour of EEG recording in this group was after the right and left side craniectomy respectively, and again always under the influence of the neurolept mixture.

SWDs were marked at visual inspection of the EEG of the right and left hemisphere independently (differential recordings between frontal and parietal cortex) based on commonly used criteria: trains of sharp spikes and slow waves lasting minimally 1 s, an amplitude of the spikes at least twice the background, frequency of the SWDs between 7 and 10 Hz and an asymmetric appearance of the SWDs [30, 31]. In case of doubt, i.e. after craniectomy or after cortical resection the monopolar recordings were used in order to decide whether a SWD was present or not. The same criteria were used for the monopolar and bipolar recordings. The SWDs seen under the neurolept-analgesic mix were visually identical to the spontaneous SWDs commonly seen in WAG/Rij rats, although the mean duration of the SWDs was longer than commonly observed [30, 32].

#### Histological verification

Immediately after euthanasia the brains of the animals were removed and fixated in formaldehyde 3% for 30 days and 30% sucrose/PBS for 4 days. Coronal slices (100 μm) were made with a microtome and stained with Cresyl violet. Three slices per animal were inspected: one from the frontal, one from the middle and one from the posterior part of the resected cortex.

#### Statistical analysis

The incidence of SWDs was determined per 30 min EEG recording. The EEG recordings in the base-line of the experimental and control group were only 24 minutes considering the duration of the first wash out period of isoflurane.

All data were statistically analyzed with SPSS 19.0. For the data of the unilateral lesion, a general linear model repeated measures ANOVA with side (left vs right) and time (baseline, post craniectomy and post unilateral lesion) as within subjects factors was used to determine the statistical significance of the main effects and their interaction. SWDs were no longer present following the removal of the second (left) focal region, and therefore statistical tests are not meaningful for these (4^th^ and 5^th^) recording hours.

Paired and unpaired t-tests were used to establish changes in the amplitude of the spike of the SWDs in both hemispheres after right craniectomy and whether these changes were different for the right and left hemisphere.

A p value of 0.05 was chosen as the threshold level for significance. Additionally, t-tests for dependent groups were used as post-hoc tests to compare side differences at different time points and differences within a hemisphere between different time points. The data of the control group were similarly analyzed as the experimental group with side (left vs. right) and time as within subjects factors.

## Results

### Experimental group

**Fig 4:**
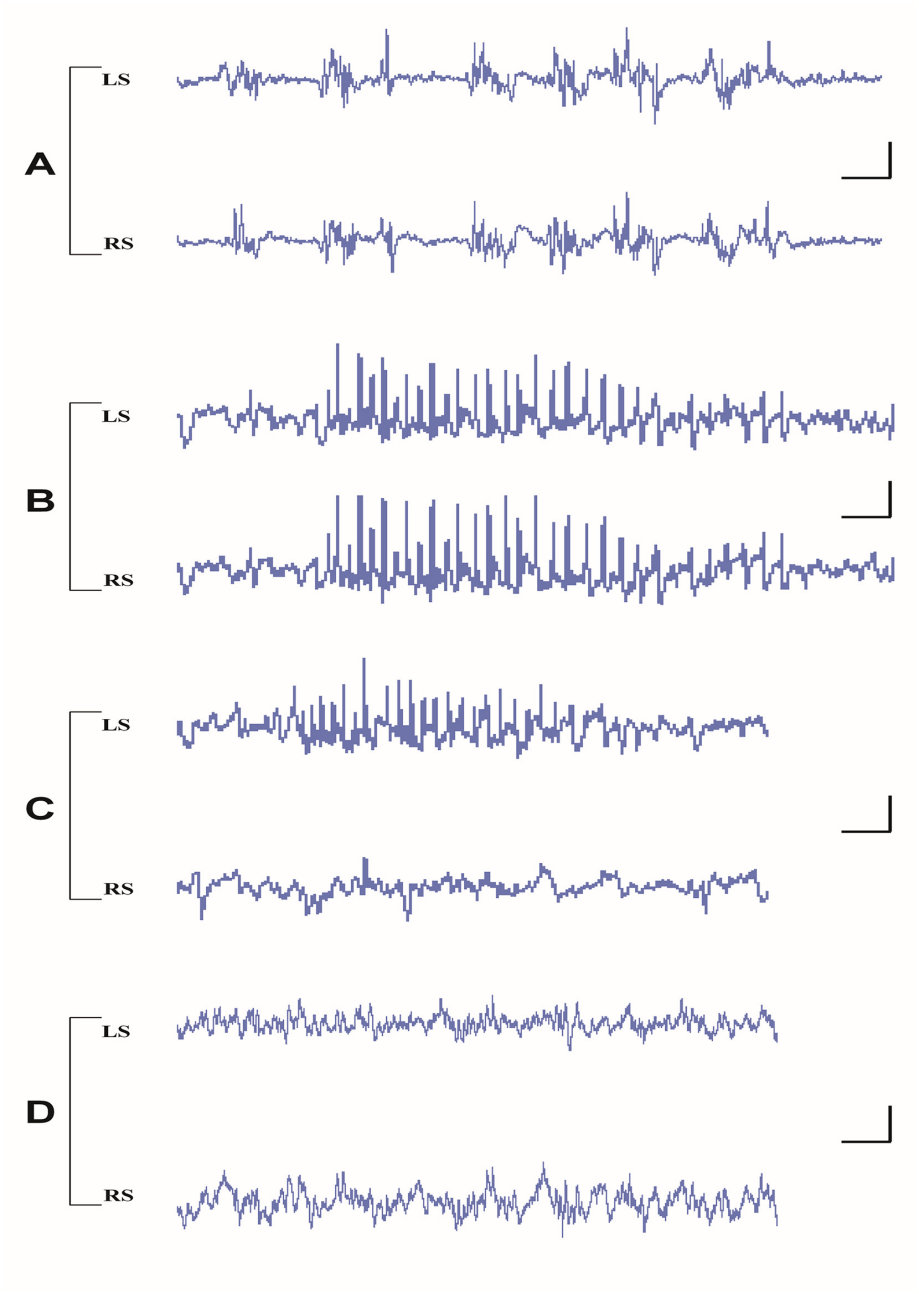
Examples of EEG recording in various phases of experiment in the two hemispheres. After systemic administration of isoflurane plus buprenorphine (A), buprenorphine plus haloperidol (B), Post unilateral right lesion (RH) buprenorphine plus haloperidol (C), and Post bilateral lesion buprenorphine plus haloperidol (D). LS = left side, RS = right side. Calibrations: vertical bar 125μV, horizontal bar: 0.625 sec.

The rats were under the influence of buprenorphine throughout the whole experiment. During isoflurane anesthesia all rats showed an EEG with burst suppression and no SWDs were noticed. The bursts appeared bilateral synchronized (Fig 4A). After the injection of haloperidol and the washout of isoflurane WAG/Rij rats exhibited bilateral normally, with respect to frequency and amplitude of the spikes, appearing SWDs (Fig 4B). However, the incidence of SWDs was higher than what can be seen in freely moving drug free rats [13, 30]. The SWD inhibiting effect of isoflurane was also present at the subsequent periods when this inhalation anesthesia was repeated at surgical intervention periods.

**Fig 5:**
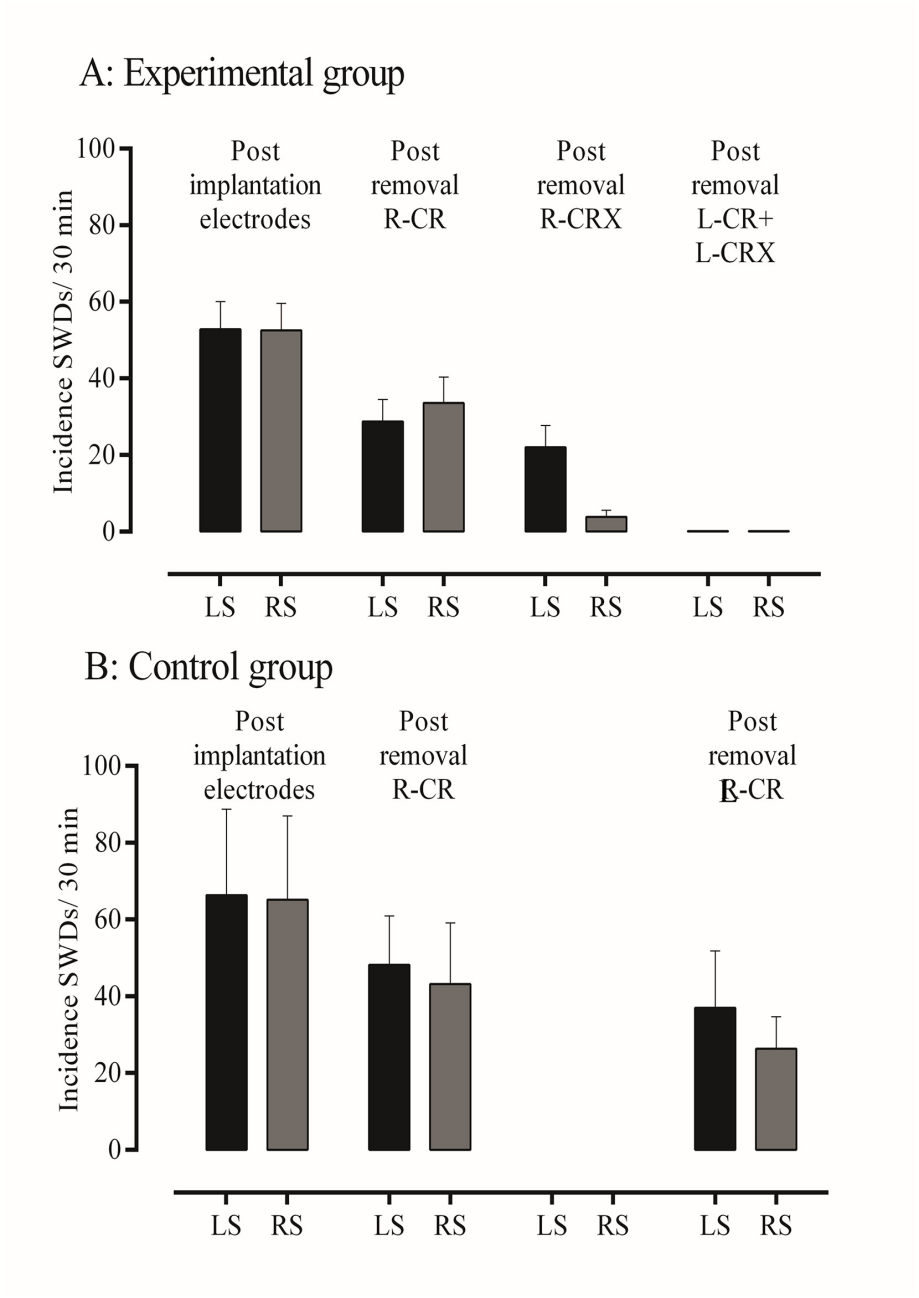
A: Incidence of SWDs (mean ± S.E.M.) per 30 min in various phases of the experiment. During base-line, after right-sided unilateral craniectomy, right-sided unilateral cortical lesions, and bilateral lesion on EEG recordings in Experimental group. The decrease from base-line to after B: Incidence of SWDs (mean ± S.E.M.) per 30 min during base-line, after right-sided unilateral craniectomy, and after left-side craniectomy in Control group. LS = left side, RS = right side.

In all rats of the experimental group, unilateral resection of somatosensory cortex affected SWDs but differentially for the two hemispheres, an example is depicted in Fig 4C. The data on the incidence of SWDs in the various phases of the experiment are given in Fig 5A. Post lesion (resection), the incidence of SWDs was unchanged in the intact hemisphere compared to post craniectomy recording period of the resected hemisphere. In contrast, SWD were rare in the resected hemisphere. The ANOVA showed significant effects for the incidence of SWDs for time (F = 11.10, df 2,26, p < .001, η^2^ = .58), left-right (F = 7.06, df 1,8, p < .03, η^2^ = .47) and their interaction (F = 10.30, df 2,16, p < .001, η^2^ = .56), post hoc t-tests showed there were no differences between left and right hemisphere before and after craniectomy, that unilateral craniectomy reduced the incidence SWDs at both sides (p < .05), while the unilateral resection of the cortex reduced SWD at the lesioned hemisphere (p < .05), but not at the intact hemisphere. Subsequent lesions of the previously intact hemisphere completely abolished all SWDs for the entire (2 hour) recording period in both hemispheres; an example of an EEG epoch following bilateral resection can be found in Fig 4D.

SWDs were no longer present, in neither the right nor left hemisphere after resection of previously intact hemisphere. The incidence of SWDs (mean ± S.E.M.) decreased from 52.8 ± 7.3 (left) and 52.6 ± 7.0 (right side)/per 30 min pre lesion to 0 on both sides.

Pearson correlation coefficients between the depth of the lesions in the frontal, middle and posterior part and the number of SWDs on both sides after the unilateral lesions were made. All correlations were small (between .33 and .03) and non significant, supporting the hypothesis that it is not the amount of resected cortical material, but the fact that lesions perse were made is a likely explanation for the diminishment of SWDs.

**Fig 6:**
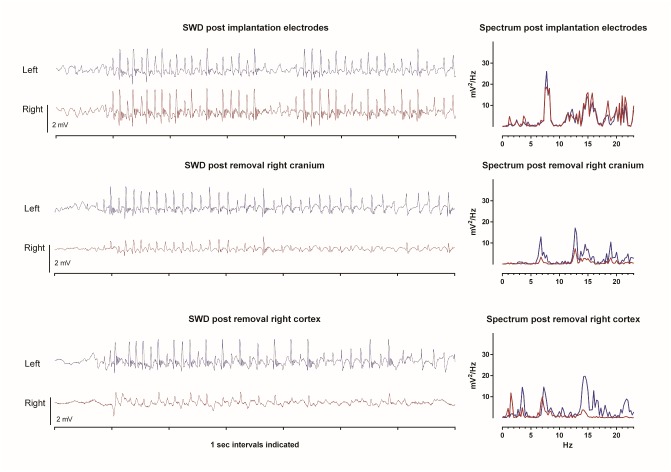
Spike-wave discharges in acute neurolept anesthetized WAG/Rij rats. Example of bilateral differential LPF recording of the left and right hemisphere and spectral plot (1–25 Hz) of a SWD in an acute neurolept anesthetized WAG/Rij rat (for details on electrode position see accompanying text) after the electrodes have been implanted (top). After removal of the right cranium, SWDs with clear spikes and slow waves are seen, albeit with a smaller amplitude both during background and during SWD (middle). The characteristic peak frequency of the SWDs in both hemispheres remains unchanged, as well the presence of its characteristic harmonics. The diminishment of the amplitude at both sides can be best appreciated from the spectral plots, the reduction is largest at the lesioned hemisphere, although the left-right difference was not statistically significant. Bottom: SWD post removal right cortex shows that SWDs are clearly visible in the intact (left) hemisphere, their identification in the right hemisphere is doubtful since they no longer fulfill the criteria of SWDs (van Luijtelaar and Coenen, 1986).

The amplitudes of the all SWDs as recorded in the right and left hemispheres in the lesioned animals were calculated in the different phases of the experiment. Examples of SWDs and their powerspectra as detrmined by a Fast Fourier analyses are presented in Fig 6. It was found that right side craniectomy reduced the amplitude of SWD on the left and right side (t-tests for paired observations, n = 9, both p’s < .05) in the experimental group by 28 and 43% respectively. Similar changes were found in the control group. However, as can be seen in Fig 6, SWDs keep their charactertistic morphology albeit with a smaller amplitude and they seem to be less regular as expressed on more peaks in the spectrogram. The size of this decrease in amplitude in the left and right side was not statistically different. Next it was found that lesions on the right side did not further decrease the amplitude on either the left (n = 9) or right (n = 5) side.

### Control group

Fig 5B depicts mean ± S.E.M of the incidence of SWDs. SWDs occurred bilaterally symmetrical and simultaneously in both hemispheres. The baseline of the incidence of SWDs as determined in 30 min before removal of the cranium was 66.3 ± 22 (left side) and 65.2 ± 22 (right side). SWDs tended to reduce gradually over time or as a consequence of craniectomy. Statistical evaluation showed neither a time effect (F = 2.24, df 2,10, p> .05, η^2^ = .31), nor a side effect (F = 3.15, df 1,5, p> .05, η^2^ = .39), although the effect sizes were rather large. This suggests that a larger sample size would yield significant main effects.

### Histological Evaluation

The extent of the cortical lesions included most of the layers of the cortex but it showed some variation between animals, details about the size of lesions in the resected animals are presented in Table 1. Photographs of lesions with different depth are presented in Fig 1C. Also there were some differences between the left and right side. In three rats (nrs 4, 5 and 6) the bilateral resection was restricted to cortical layers II-III and IV of the frontal, middle and posterior part, while the other layers of the cortex were fully intact. In four rats (nrs 1, 2, 7 and 8), the bilateral resection of the somatosensory cortex was larger, layer V was removed at least in one location, in rat nrs 3 and 9 the resection was extensive until layer VIa in at least one of the three sections. The corpus callosum did not show any damage in any of the rats, as could be inferred from microscopic inspection.

**Table 1:** Histological verification of the bilateral resected areas in 9 WAG/Rij rats. The numbers I to VIa represent the cortical layers that were removed constituting the somatosensory cortex. LS = left side, RS = right side.

HISTOLOGICAL VERIFICATIONS						
RAT	SOMATOSENSORY CORTEX					
	Frontal Part		Middle Part		Posterior Part	
	LS	RS	LS	RS	LS	RS
Rat 1	II-III	IV/V	II-III	V/VIa	II-III	IV
Rat 2	IV	IV	V	IV	V	IV
Rat 3	V/VIa	V/VIa	V	V	V	V
Rat 4	II-III	IV	II-III	IV	II-III	IV
Rat 5	II-III	IV	II-III	IV	II-III	IV
Rat 6	IV	IV	IV	IV	II-III	IV
Rat 7	IV	V	IV	V	IV	V
Rat 8	IV	V	IV	V	II-III	IV
Rat 9	V/VIa	V/VIa	V/VIa	V	V	V

## Discussion

The major outcomes of this acute study in neurolept anesthetized WAG/Rij rats are that a unilateral lesion of the assumed focal region decreased the incidence of SWDs and this reduction was different in the two hemispheres. Bilateral resection completely abolished all SWDs.

### Removal of foci or interference with a network

It is generally assumed that SWDs in rodents take place in an interconnected intact cortico-thalamo-cortical network, although the exact interactions between the cortex and different thalamic nuclei necessary for the generation and maintenance of SWDs are not fully understood [9, 14, 33]. It is clear that SWDs in WAG/Rij rats are initiated in the perioral region of the somatosensory cortex (S1po) [11,15], most likely by neurons located in the deep cortical layers, as was established in GAERS [12]. From this point the early appearance of SWD-activity can be easily visualized by local field potentials from the depth of the somatosensory cortex [15, 34]. Our present data demonstrate that removal of the cortical regions which contain the initiation site of the SWDs (the foci) reduces SWDs in both hemispheres. Even when the focal layers, i.e. the cortical layers V and VI, are still intact, SWDs are reduced, suggesting that a decreased intactness of the cortical columns, part of the neural circuits in which SWDs are initiated, spread and maintained is responsible for a reduction or complete abolishment of SWDs. This conclusion is also supported by the lack of significant correlations between SWD incidence and the depth (size) of the cortical lesions.

Other studies aiming to test the role of various parts of the network in their contribution to the occurrence of SWDs have shown that SWDs are suppressed by a functional inactivation of the whole neocortex by inducing a spreading depression in GAERS [35] or by micro-infusion of local inactivating drugs such as phenytoin in the subgranular layers and Lidocaine at the surface of the S1po in WAG/Rij rats [23, 36]. Moreover, micro-infusion of ethosuximide in the region S1po, again in GAERS, causes a full and immediate decrease in SWD number, comparable to that tested after systemic administration of the same drug, supporting the involvement of this area as a crucial and specific area in the initiation or occurrence of SWDs [22, 24, 37]. Similarly, inactivation studies of various parts of the lateral thalamus including the rostral RTN abolished SWDs both in GAERS, as in WAG/Rij rats [38, 39, 40, 41, 42], suggesting that an interference with the intactness of this circuitry is crucial for the diminishment of the occurrence of SWDs.

### Unilateral lesions: a differential reduction in the ipsilateral and contralateral hemisphere

The corpus callosum is the principal anatomical structure, necessary for the bilateral synchronous cortical and thalamic SWDs in intact brains since callosal transsections reduced the left right co-occurrence. It seems that each hemisphere is able to initiate SWDs independently [35]and that SWDs quickly appear bilateral symmetrically through the interhemispheric monosynaptic projections of the callosal projecting neurons [43]. The interhemispheric connections of the homotopic regions of the somatosensory cortex are constituent part of the corpus callosum [44]. Both a network analyses and Diffusion Tension Imaging study showed the relevance of the cortico-cortical interhemispheric connections for SWDs between the left and right somatosensory cortices in these absence epileptic rats [45, 46]. About 80% of the cell bodies of these callosal projecting neurons in rodents principally reside in cortical layers II/III, about 20% in layer V and a small fraction in layer VI [47]. Layers I through III are the main target of interhemispheric cortico-cortical afferents, and layer III is the main source of cortico-cortical efferents [48]. The unilateral resected focal region is no longer able to initiate SWDs. However, information transfer from the intact hemisphere via the corpus callosum to the partial resected hemisphere is still possible. This allows the presence of some SWDs in the resected hemisphere. It is also possible that SWDs, initiated at the intact hemisphere involve the contralateral hemisphere via interhemispheric thalamic projections. The reticular thalamic nuclei are known to project to the contralateral thalamus through bilateral connections with the ventro medial nuclei of the thalamus and intralaminar nuclei and can influence the activity of wide territories of the cerebral cortex and basal ganglia of both hemispheres [49]. It is clear that callosal and interthalamic transsections studies are necessary to establish the role of the contralateral hemisphere after ipsilateral lesions.

The differential effects of unilateral lesions, as revealed by the significant interaction between left-right and pre-post lesion shows that the occurrence of SWDs in the left and right hemispheres should not be considered as completely independent processes. Instead, our data show that the effects of an unilateral lesion exert a larger effect at the ipsilateral than on the contralateral side. Although it seems logical that SWDs generated in one hemisphere quickly involve the other hemisphere through the excitatory pathways of the callosal projecting neurons interconnecting the focal regions in the left and right hemisphere [43, 45, 50], it also seems that the intact hemisphere is no longer inhibited by the resected hemisphere and that the number of SWDs are higher at the intact side as compared to the number at the lesioned side. This proposal would not be against the view that the function of interhemispheric transfer of information could be both inhibitory and excitatory in the same corpus callosum [51].

### Bilateral lesions abolish all SWDs

The third main finding is that bilateral resection of the assumed cortical foci in the somatosensory cortex abolished all SWDs, although the lesions were not always extended to the deepest cortical layers. The histological examination of the size of the resections in the current experiment showed that in rats of the experimental group the resection has been done till layers III-V of the S1po and, and in only 2 animals until layer VI.

The sensory cortex including the somatosensory cortex with its S1Po is not only part of a larger cortico-thalamo-cortical and inter hemispheric network for information transfer, it is also columnar organized with many connections between various layers within the thickness of the cortex. In every layer morphological subtypes of cells are present [52, 53], which project to various cortical regions [54]. Next, excitatory inputs from layer IV to supra granular layers III and II regulate and even amplify the sensory information transcolumnar [55], whereas projections from layer III to layer V and VI are also involved in intracolumnar circuits [56].

Our results suggest that interference of inter or intra layer communication of only the superficial cortical layers and thereby altering the normal cortical signal processing is sufficient to interfere with the occurrence of SWDs. Layers IV, V and VI are responsible for the communication between cortex and thalamus, layer IV is the main target of the thalamo-cortical afferents, as well as intra-hemispheric cortico-cortical afferents [56]. The infragranular layers V and VI establish a very precise reciprocal interconnection between the cortex and the first order thalamic neurons and higher order nuclei [57, 58, 59, 60]. Interestingly, lesions of the cortex that communicated most directly with the thalamus and of cell layers that contain the most hyperexcitable cortical cells [12] involved in SWD generation, are not necessary for interference with SWD occurrence. More precise, our study points out that lesioning of the superficial layers is sufficient to prevent the occurrence of SWDs. Some support for the view that also the superficial cortical layers are also involved in the occurrence of SWDs is obtained from the Kandel and Buzsáki study [61]. These authors found sinks and sources during SWDs in all cortical layers, suggesting that also inter layer communication is necessary for the occurrence of SWDs. In addition, the basically different types of neurons present in every single column of the cortex are involved in the communication between cortical layers [53, 55, 62, 63]. In all, it does not seem necessary to resect the cortical tissue completely to abolish the SWDs in these genetic epileptic rats.

### Control group

The control group was added to our protocol in order to demonstrate the presence of SWDs during the various regimes of anesthesia both before and after unilateral and bilateral craniectomy. The analyses of the EEG recordings of control WAG/Rij rats showed that SWDs were abundantly present in all phases of the experiment and that there were no differences in parameters of SWDs between left and right side. The apparent decrease of SWDs over the recording hours as seen in the experimental and control group (Figs 5 and 6) is due craniectomy, and or to the cumulative effects of isoflurane over time or both. It has been demonstrated that craniotomy reduces the brain’s excitability for an extended period [64]. and physical stimulation of the cortex in the form of pinpricks induces a spreading depression suppressing SWDs for 1 to 2 hours [65]. It is thought that even a careful brain operation might have short term consequences on cortical excitability causing SWDs to diminish. Isoflurane anesthetic was used repeatedly and intermittently (drilling holes, removal of cranium, removal of cortical tissue) and SWDs were never seen under isoflurane anesthesia. We noticed that the recovery time of isoflurane as measured by the reappearance of the SWDs increases from about 23 min from the first discontinuation of anesthesia, to about 35 min from the 2^nd^ period of isoflurane anesthesia. It is therefore thought that both factors, craniectomy and isoflurane, contribute to the simultaneous reduction of SWDs in the left and right hemisphere over time. However, SWDs remained present in our anesthesia regime on either side.

### Is the site of the lesion crucial?

It would be interesting to make similar resections, or to make small lesions in other parts of the cortex in order to establish whether cortical lesions in different parts of the cortico-cortical network are also sufficient to prevent seizure occurrence since it might be thought that the decrease after the cortical resections is due to a non-selective effect of interfering with the functional integrity of the cortex. Polack et al. [66] established in GAERS that the blockade of neuronal activity by the topical application of the sodium channel blocker tetrodotoxin in the motor cortex did not affect the occurrence of SWDs in the somatosensory cortex, while the functional deactivation of neurons in the facial area of the somatosensory cortex by the same method abolished all ictal activities in the somatosensory cortex, including the SWD. This Polack et al. study [66] is the primary evidence that it matters for SWD occurrence which part of the cortex is inactivated or removed.

Similarly, we previously established that rostral thalamic lesions in WAG/Rij rats abolished cortical SWD, while caudal thalamic lesions enhanced SWDs [42], again demonstrating that the effects of in this case thalamic lesions regarding their SWD reducing effects are specific for the location within the cortico-thalamo-cortical network and therefore this abolishment should not be considered as being caused by a non-specific lesion effect.

The outcomes of two pharmacological studies confirm that selectivity of the somatosensory cortex as the initiation side for SWDs: infusion with ethosuximide or AMPA antagonists was only effective when applied in the somatosensory cortex and not in the motor cortex [22, 67]). Additionally, a diminishment of SWD is not very likely in case of lesions in for example the visual cortex considering that the focal facial region receives necessary input from the VPM [68] and projects back to the posterior nucleus, RTN and somatosensory thalamus and not to the visual thalamus. The visual cortex and its thalamic counterpart, the lateral geniculate, are, to the best of our knowledge, not part of the SWD generating system. Moreover, no other SWD initiating sites have been described in this genetic rodent absence model outside the somatosensory cortex. In all, a non-specific effect of the lesion is not a likely explanation for the complete abolishment of SWD after bilateral cortical lesions.

### Concluding remarks

The outcomes of the present study contribute to the discussion about the generalized nature of absence epilepsy; the successful removal of an assumed focal zone has been an argument for the distinction between focal and generalized epilepsies. Here the bilateral removal of the assumed focal regions, or more precise, a partial removal of the cortical zone dorsal to the assumed focal origin and or interference with the columnar intracortical networks was enough for complete seizure abolishment in this acute study. It is also thought that the site of the cortical lesion is specific.

It is acknowledged that craniectomy and surgical resection is a radical surgical technique for seizure inactivation. More subtle alternative techniques should be explored. Small implanted electrodes for example, would allow making selective lesions in brain tissue. Moreover, before making lesions, these electrodes might be used for local field potential recordings of SWDs and for local evoked potentials elicited by stimulation of specific afferent pathways. In this way the cortical area would be functionally mapped, the excitable focal area could be identified before making the lesions.

The experiments were carried out in anesthetized WAG/Rij rats. Although the SWDs as seen under this type anesthesia closely mimic the spontaneous occurring SWDs (Fig 6, upper trace), it is necessary to repeat these experiments in free moving animals and evaluate the long term effects of surgical manipulations. Finally, the possibility exists that surgical ablation restricted to the most superficial layers hampers the functionality of the deep layers of the cortex, considering that the intracolumn information flow is bidirectional and that resection of the dendritic arborescence is likely to modify the deep neurons integrative properties. Therefore, and as is the case in all in vitro studies and in some ablation studies in vivo, the functionality of the remaining neural tissue can be questioned. Differential recorded local field potentials measured in frontal and parietal cortex (the removed area was in between the two active EEG electrodes) showed a diminshment of the amplitude in both the experimental and control group, however no changes in amplitude of the SWDs before and after the cortical resection in either the lesioned and intact side were found, suggesting that after the lesion the remaining tissue was still good enough to generate some SWDs.

The outcomes of our study emphasize the necessity of an intact cortical circuit for the occurrence of SWDs and demonstrate that absence epilepsy is a network type of epilepsy since interference within the network involved in the communication between supra and subgranular layers disrupts seizures. SWDs also have a cortical focal origin in patients with absence epilepsy [18, 69, 70, 71, 72], although the location of their foci might be different from that in animals [73, 74]. Some of the absence epileptic patients are cognitively impaired and this depends on the anatomical site of seizure onset, the hemisphere involved and the dimension of epileptogenic area [75]. Some forms of absence seizures have their origin in the frontal lobe, especially in the mesial frontal region [76, 77] and patients with absence seizures with clear focal abnormalities on EEG were identified [78]. Since some of these patients are intractable by current medications [79], partial inactivation with modern techniques of the assumed focus or interference with an intracortical circuit might be a treatment option in these refractory patients. Moreover, seizure control is of utmost importance because it might restrict cognitive damage since the duration of refractory epilepsy is a major determinant of cognitive deterioration, affecting also quality of life [80, 81, 82]. These first experimental outcomes of cortical resection teach us that surgical techniques in combination with electrophysiological recordings should not be rejected at forehand although there are many questions whether the present acute results in this genetic absence model can be translated to a chronic preparation and later to patients without further functional compromising patients.
